# Strategies to Overcome Barriers to Implementation of Alcohol Screening and Brief Intervention in General Practice: a Delphi Study Among Healthcare Professionals and Addiction Prevention Experts

**DOI:** 10.1007/s11121-016-0653-4

**Published:** 2016-05-11

**Authors:** L. Abidi, A. Oenema, P. Nilsen, P. Anderson, D. van de Mheen

**Affiliations:** Department of Health Promotion, School of Public Health and Primary Care, Maastricht University, PO Box 616, 6200 MD Maastricht, The Netherlands; Department of Medical and Health Sciences, Linköping University, Linköping, Sweden; Institute of Health and Society, Newcastle University, Newcastle, UK; IVO Addiction Research Institute, Rotterdam, The Netherlands; Erasmus Medical Center, Rotterdam, The Netherlands

**Keywords:** Alcohol, Screening, Brief intervention, Implementation, General practice

## Abstract

Despite the evidence base, alcohol screening and brief intervention (ASBI) have rarely been integrated into routine clinical practice. The aim of this study is to identify strategies that could tackle barriers to ASBI implementation in general practice by involving primary healthcare professionals and addiction prevention experts. A three-round online Delphi study was carried out in the Netherlands. The first-round questionnaire consisted of open-ended questions to generate ideas about strategies to overcome barriers. In the second round, participants were asked to indicate how applicable they found each strategy. Items without consensus were systematically fed back with group median ratings and interquartile range (IQR) scores in the third-round questionnaire. In total, 39 out of 69 (57 %) invited participants enrolled in the first round, 214 participants completed the second round, and 144 of these (67 %) completed the third-round questionnaire. Results show that participants reached consensus on 59 of 81 strategies, such as the following: (1) use of E-learning technology, (2) symptom-specific screening by general practitioners (GPs) and/or universal screening by practice nurses, (3) reimbursement incentives, (4) supportive materials, (5) clear guidelines, (6) service provision of addiction care centers, and (7) more publicity in the media. This exploratory study identified a broad set of strategies that could potentially be used for overcoming barriers to ASBI implementation in general practice and paves the way for future research to experimentally test the identified implementation strategies using multifaceted approaches.

**Electronic supplementary material:**

The online version of this article (doi:10.1007/s11121-016-0653-4) contains supplementary material, which is available to authorized users.

## Background

Alcohol consumption is a wholly or contributory cause for more than 200 diseases, injuries, and other health conditions (WHO 2014). Globally, alcohol is the fifth most important risk factor for ill health and premature death (Lim et al. 2012). In the Netherlands, more than 10 % of the population of 16- to 69-year olds reported alcohol-related problems and drinking alcohol at levels considered problematic (Van Dijck and Knibbe 2005). Early detection and early treatment can have an important impact on reducing the detrimental effects of problematic alcohol use. By detecting and assisting those with “risky” or “problematic” alcohol use at an early stage, healthcare professionals can deliver brief interventions aimed at increasing patients’ confidence and ability to change their drinking behavior by providing information, feedback, health education, skill building, and practical advice (McCormick et al. 2010). The scientific literature has provided robust evidence that supports the efficacy and (cost-) effectiveness of alcohol screening and brief interventions (ASBI) in primary healthcare settings (Bertholet et al. 2005; Kaner et al. 2009), indicating that ASBI, compared with control conditions, leads to significant reductions in alcohol consumption (average reduction of 38 g a week) as well as savings to healthcare resources (Angus et al. 2014; Purshouse et al. 2013). However, translating scientific knowledge into sustained widespread implementation of ASBI in routine primary healthcare has proven to be difficult despite many implementation efforts.

A previous line of research has identified barriers which are related to difficulties with ASBI implementation such as a lack of knowledge about early symptoms of problematic alcohol use (Reinholdz et al. 2011), or lack of knowledge about risk groups (e.g., elderly patients, patients suffering from psychological disorders, young patients with conduct problems) (Boomsma et al. 2014). Additionally, a lack of time, a lack of resources (Damschroder and Hagedorn 2011; Johnson et al. 2011), and even practitioners’ own alcohol use (Burgering and Willems 2013) have been identified as barriers as well. Although multifaceted implementation strategies targeting provider, patient, and organization factors are considered to be most effective to implement ASBI (Anderson et al. 2004), most studies have found small effects (Funk et al. 2005; Van Beurden et al. 2012). Furthermore, sustainability of implementation over time has been found to be low, which may be due to a failure to match implementation strategies to barriers that are relevant for the continuation of an innovation (Wensing et al. 2014).

Translational researchers are acknowledging more and more that an accumulation of positive evidence is not sufficient to achieve widespread implementation of an intervention in clinical care (Lean et al. 2008). Next to an evidence-based approach, there is a need for a practice-based approach in which ASBI is adapted to local circumstances so that it can be implemented in an efficacious and sustainable fashion (McCormick et al. 2010). For instance, in three case studies of ASBI implementation, research conducted in England, New Zealand, and Catalonia, McCormick et al. (2010) provided real-world evidence in support of matching ASBI to the local context and with the skills health professionals already used in practice. The following general principles emerged from their results: tailoring procedures to fit with local circumstances, and breaking the process down into clinically acceptable steps and negotiating where there is flexibility.

Fitting ASBI to the local context requires that academics, healthcare practitioners, and addiction prevention experts work together to identify pragmatic implementation strategies (Kaner 2010). The current ASBI literature, however, does not provide any guidance on which implementation strategies are most appropriate or most *applicable* from health professionals’ and addiction prevention experts’ points of view. Moreover, exploring consensus and differences between health professionals’ and addiction prevention experts’ gives a more in-depth picture of requirements and strategies needed to implement ASBI in general practice team settings. Therefore, the aims of the current study are to (1) identify applicable strategies to overcome known barriers as suggested by health professionals and addiction prevention experts, and to (2) identify the extent to which health professionals and addiction prevention experts agree on the applicability of the identified implementation strategies.

## Methods

We conducted an online three-round Delphi study in the Netherlands, using the online form management system, Formdesk (Innovero Software Solutions BV). The Delphi methodology is a technique for systematic structuring of informed opinions from a large group of experts on complex issues by means of iteration with controlled feedback (Linstone and Turoff 1975). The Delphi method has been used before in implementation research informing the use of implementation strategies on a range of topics (Heather et al. 2004; Huijg et al. 2013; Maijala et al. 2016).

### Theoretical Framework

The behavior change wheel (BCW) is a theoretical framework which provides a comprehensive method of classifying and choosing strategies and policies most likely to be effective in influencing behavior change (Michie et al. 2011). At the center of the BCW are three components: capability, opportunity, and motivation which interact to determine behavior (i.e., the COM-B system). The framework distinguishes intervention functions that can be used to facilitate behavior change (i.e., education, persuasion, incentivization, training, enablement, coercion, restriction, environmental restructuring, and modeling). Moreover, to support the intervention functions, the BCW links the intervention functions to seven policy measures (e.g., guidelines, fiscal policies, communication/marketing, regulation, legislation, environmental/social planning, and service provision) (Michie et al. 2011). In order to provide a comprehensive picture of intervention functions and policies which are applicable for ASBI implementation in general practice, proposed strategies by health professionals and prevention experts will be categorized as intervention functions or policy measures as distinguished in the BCW.

### First Round

The aim of the first round was to obtain a list with new ideas and workable solutions to known barriers (defined under questionnaire) of ASBI implementation.

#### Participants and Procedure

In collaboration with the Department of Family Medicine of Maastricht University and Mondriaan mental healthcare institute, a list of 69 potential participants was compiled consisting of 45 health professionals (i.e., general practitioners (GPs), practice nurses), and 15 addiction prevention experts (i.e., addiction prevention workers, academic researchers, and managers of addiction prevention departments) (Table 1). Addiction prevention experts and managers of addiction prevention departments in the Netherlands work in collaboration with primary healthcare centers to support them in prevention work. Therefore, their opinion about what they can offer general practices in terms of preventative services, including ASBI, and how these services should be effectively implemented, should be taken into account. Academic researchers, who are experts in implementation of innovations in primary healthcare, are often involved in practical implementation in the Netherlands. Moreover, they might draw ideas from overlapping research fields which successfully implemented interventions in primary healthcare. Potential participants were approached via e-mail or telephone. The process of the Delphi study was explained, and an invitation was issued to participate in all rounds. All participants received an online questionnaire in which barriers to ASBI implementation as identified in research were explained. Participants were asked and encouraged to come up with ideas and solutions to the presented barriers. Invitees received a reminder 3 weeks after the first invitation and had a period of 3 weeks to complete the first-round questionnaire.

**Table 1:** Response rates

		Round 1		Round 2^a^	Round 3	
		*N*	*N* (%)	*N*	*N*	*N* (%)
		Invited	Response	Response	Invited	Response
Health professionals	GP	32	13 (40)	60	60	37 (62)
	Practice nurse—mental health care	17	11 (64)	83	83	63 (76)
	Practice nurse—somatic care	5	4 (80)	12	12	6 (50)
	Psychologist	0	0 (0)	1	1	0 (0)
Addiction prevention experts	Addiction prevention worker	9	6 (66)	50	50	30 (60)
	Researcher	2	2 (100)	3	3	3 (100)
	Manager prevention department addiction center	4	3 (75)	5	5	5 (100)
	Total	69	39 (57)	214	214	144 (70)

^a^Due to open recruitment methods (e.g., advertisements), round 2 invitation rates were incalculable

#### Questionnaire

The first-round questionnaire was developed through an analysis of the target behavior: ASBI delivery. Barriers to ASBI delivery behavior reflecting *capability*, *motivation*, or *opportunity* (COM-B) components were identified in the literature (Burgering and Willems 2013; Johnson et al. 2011; Wilson et al. 2011): (1) lack of knowledge (capability), (2) use of personal reference frames to discuss alcohol (capability), (3) lack of motivation to work with problem drinkers (motivation), (4) uncertainty about professional role in working with problem drinkers (motivation), (5) lack of time (opportunity), (6) lack of incentives (opportunity), (7) lack of low-threshold referral options (opportunity), (8) lack of cooperation with addiction centers (opportunity), and (9) difficulty in addressing the subject of alcohol use (opportunity). Based on these barriers, open-ended questions were formulated for the first-round questionnaire (Table 2). Each question included a short explanation of the barrier and instructions to think about and report possible solutions to the barrier. A section was also included to allow participants to provide additional comments if they so wished. The questionnaire was pretested with two GPs, two academic researchers, and an addiction prevention worker and was adjusted accordingly.

**Table 2:** Questions based on barriers identified in literature and categorized in the COM-B system (Michie et al. 2011)

COM-B	Barriers	Questions
Capability	Lack of knowledge	1. What is needed to increase knowledge about symptoms, risk groups and intervention techniques to effectively implement ASBI in routine practice?
	Use of personal reference frames to discuss alcohol	2. What is needed to discuss alcohol use with patients independent from reference frames formed by own alcohol use?
Motivation	Lack of motivation	3. What is needed to increase motivation to work with problematic alcohol users?
	Lack of incentives	4. Which incentives are needed to implement ASBI effectively in routine practice?
	Uncertainty about professional role	5. What is the role of the GP/practice nurse in screening and brief intervention for patients with problematic alcohol use in GP practices?
Opportunity	Difficulty and sensitivity of subject	6. What is needed to make the subject “alcohol use” easier to discuss for health professionals in general practice?
	Lack of time	7. What is needed to implement ASBI in routine care despite lack of time?
	Lack of low-threshold referral options	8. What is needed to utilize low-threshold referral options in general practice?
	Lack of collaboration with addiction treatment centers	9. What is needed to improve collaboration with addiction treatment centers?

#### Analyses

Participants’ responses were listed to form an extensive inventory of potential strategies to overcome each barrier. A content analysis was conducted to group similar answers together, eliminate duplicate responses, and include unique responses. First-round responses were listed as items for the second-round questionnaire.

### Second Round

The aim of the second round was to reach consensus among the participants on the applicability of the strategies to overcome barriers to ASBI implementation.

#### Participants and Procedure

In the second round, participants were asked to rate each item, i.e., implementation strategy, in the questionnaire on applicability of the strategy. First, all participants of round 1 were approached again to participate in the second-round questionnaire. Additional GPs, practice nurses, and addiction prevention experts were recruited by means of advertisements on healthcare organizations’ Web sites with information about the study and a weblink to the online questionnaire. Academic researchers in the field of addiction prevention in primary healthcare as well as general practices throughout the Netherlands were also contacted by telephone or by e-mail. The process of the Delphi survey was explained, and those who enrolled in the study received the weblink to the online questionnaire via e-mail. In addition, a snowball sampling technique was used in which participants were asked to suggest others who might be interested in participating in this study. Managers of 12 addiction prevention departments of addiction centers in the Netherlands were asked if they would invite their employees to participate in this study. All invitees received a reminder 3 weeks after the first invitation and had a period of 3 weeks to complete the second-round questionnaire.

#### Questionnaire

The second-round questionnaire (supplementary table: available online) was structured around the various barriers that had been identified. Each barrier was first briefly introduced, after which the potential strategies were provided. Participants were asked to indicate to what extent they found the solution applicable, using a seven-point Likert scale with response categories ranging from “1” (very inapplicable) to “7” (very applicable). Given the fact that there was variability in preferences for types of screening (e.g., symptom-specific screening, universal screening) in the first-round responses, a question about screening methods was added to the questionnaire. Questions concerning the role of GPs/practice nurses ranged from “1” (strongly disagree) to “7” (strongly agree).

#### Analyses

Median scores were calculated to determine the extent to which participants found the item under consideration applicable, with a median score of ≥6 indicating an “applicable” or “very applicable” strategy. Interquartile range (IQR; distance between the 25th and the 75th percentiles) scores were calculated to determine the degree of consensus between participants on each item. An IQR score of ≤1 signifies that at least 50 % of the participants deviate one point or less from one another and is generally regarded as indicative of a high degree of consensus. All statistical analyses were conducted with SPSS version 20.

### Third Round

In line with the Delphi methodology, the aim of the third-round questionnaire was to explore whether further consensus could be reached on items for which no consensus was obtained in the second round and to explore remaining differences between groups.

#### Participants and Procedure

All participants who had completed the second-round questionnaire were invited by email to complete the third-round questionnaire. Invitees received a reminder 3 weeks after the first invitation and had a period of 3 weeks to complete the third-round questionnaire.

#### Questionnaire

The third-round questionnaire included those questions from the second-round questionnaire on which no consensus was reached. The median ratings and IQR scores derived from the second round were provided for each item to allow participants an opportunity to rethink their answer in light of the previous results and increase consensus among participants. The participants were asked to re-rate items on the same seven-point Likert scale in the light of the group median and IQR score of each item.

#### Analyses

Attrition analyses from rounds 2 and 3 were conducted. Median and IQR scores were calculated, and the Kruskal–Wallis test was used to test whether groups of experts differed significantly from each other in opinion about the applicability of items on which no consensus was reached after this round (Table 3). Items on which the groups differed were further explored by post hoc pairwise Mann–Whitney *U* tests to investigate which groups differed from each other (Table 4).

## Results

### First-Round Results

Thirty-nine out of 69 (57 %) invited participants enrolled in the first round (Table 1). The first round generated 81 closed-ended items for the second-round questionnaire (supplementary table: available online). Most identified strategies can be classified in the following BCW intervention functions as described in the “Methods” section: (1) education, (2) training, (3) environmental restructuring, (4) enablement, (5) incentivization, (6) modeling, (7) persuasion, and the following BCW policy measures: (8) guidelines, (9) regulation, (10) service provision, and (11) communication/marketing (Michie et al. 2011).

### Second- and Third-Round Results

In total, 214 participants completed the second round and 144 of these (67 %) completed the third-round questionnaire. An overall test of attrition bias showed that dropout is equally distributed among gender and age. Those who remained in the study in round 3 were not more likely to be of a certain profession (i.e., 60 % of addiction prevention experts, 62 % of GPs and 63 % of practice nurses who completed round 2 also completed round 3), nor was previous response type related to dropout.

Total group results from the second round showed that participants reached consensus (IQR ≤ 1) on 50 items. After receiving feedback about the second-round results, participants reached consensus on another nine items in the third round, making a total number of 59 items. The 59 consensus items were all rated high on applicability. The categorization of strategies in intervention functions or policy measures will be presented in parentheses in the following section.

- *Q.1. What is needed to increase knowledge about symptoms*, *risk groups and intervention techniques to effectively implement ASBI in routine practice*? First, strategies targeting the provider that had achieved consensus and a high rating on applicability included the following: “supportive materials such as Web sites” (enablement), “following expertise-enhancement training” (training), “an educational intervention through E-learning” (education), and “learning through examples and insights into favorable results of ASBI” (education). The following strategy related to general awareness and the GP setting was consensually endorsed: “more publicity and attention in the media and in the general practice setting” (communication/marketing). No consensus was reached about “the applicability of enhancing knowledge by means of using an app” (enablement), “involving an addiction consultant in the general practice setting” (service provision) or “involving addiction centers in the organization of information meetings for GPs and practice nurses” (service provision).
- *Q.2. What is needed to discuss alcohol use with patients independent from personal drinking norms and reference frames*? Three items were consensually supported on applicability: “standardizing discussing alcohol through clearer guidelines”; “protocols and norms” (guidelines); “peer-to-peer coaching about professional attitude to become more aware of own reference frames, alcohol norms, and behavior” (training); and “destigmatization of problematic alcohol use” (communication/marketing). No consensus was reached on the following items: “giving GPs and practice nurses more information about alcohol usage and alcohol norms of peer health professionals” (education) and “discussing alcohol usage of GPs and practice nurses in training” (training).
- *Q.3. What is needed to increase motivation to discuss alcohol use with patients*? Consensus was reached on the applicability of a wide variety of strategies to increase motivation to work with problematic alcohol users. Four items at the provider level emphasized the importance of education: “knowledge about how to work with problematic alcohol users,” “clear instructions for treatment,” “more insight into how symptoms are associated with problematic alcohol use,” and “more insight into the effectiveness of ASBI.” Furthermore, there was consensus about the applicability of supportive materials (enablement): “practical tools for patients (e.g., alcohol diary or agenda)” and “distinguishing problematic alcohol users from dependent drinkers” (enablement). At the organizational level, three items about referral options emphasized the importance of low-threshold accessibility and publicity: “more accessible referral options and consultations with experts for support and cooperation” (service provision) and “more publicity about the possibilities of ASBI by means of E-health” (communication/marketing, education). However, there was little consensual support for “financial incentives for ASBI” (incentivization) or “learning to establish trust between health professional and patient” (education) as ways to increase motivation to discuss alcohol use with patients.
- *Q.4. Which incentives are needed to effectively implement ASBI in routine practice*? Four out of seven items achieved consensus on applicability of incentives: “more insight into the health profits of ASBI for patients” (persuasion), “financial reimbursements from health insurance companies to implement ASBI” (incentivization), “more financial contributions to projects in general practice about problematic alcohol use” (incentivization), and “implementing a practice nurse specialized in addiction problems without extra costs” (service provision). In contrast, no consensus was reached on the following items as incentives to implement ASBI: “more insight into the financial profits of ASBI” (persuasion), “faster referral and treatment in primary care and secondary care” (service provision), and “a monetary fee per screened patient” (incentivization).
- *Q.5. What is the professional role of the GP*/*practice nurse in ASBI*? The GP, the practice nurse mental healthcare, and practice nurse somatic care were all considered to have important roles in the early detection of problematic alcohol use and this item showed consensus around high agreeability. Healthcare providers’ role in brief treatment was, however, less clear-cut: participants agreed and consented on the role of the GP in providing brief advice/monitoring and motivational interviewing, but participants agreed less and failed to reach consensus about the role of the practice nurse somatic care in brief treatment of problematic alcohol use.
- *Q.6. What is needed to implement ASBI in routine care despite lack of time*? Four items with consensus around high applicability concerned specific time-saving methods of ASBI delivery: “adding a question about alcohol to a frequently used questionnaire, such as the “Four-Dimensional Symptom Questionnaire (4DSQ)” (enablement), “using a short and simple screening instrument such as the AUDIT-C” (enablement), “giving patients a self-report questionnaire” (enablement), and “implementing a short questionnaire in the registration system” (environmental restructuring). Another two items with consensus around high applicability focused on the individual level: “increasing knowledge about the fact that a short intervention costs little time and can be effective” (education) and “if one suspects problematic alcohol use, scheduling a second appointment with the patient” (regulation). Also consensually endorsed was the need for more time per consultation (regulation). No consensus was reached on the following three items which concern more structural changes at the organizational level of the general practice setting: “implementing online programs for diagnosis and treatment plans” (environmental restructuring), “implementing an alcohol consultation” (service provision), “distribution of self-report questionnaires by receptionists in the waiting room before consultation” (enablement), nor was consensus reached for “more financial aid for conducting ASBI” (incentivization).
- *Q.7. What is needed to utilize low*-*threshold referral options in general practice*? Seven items received consensual support to enhance utilization of low-threshold referral options: “providing general information and publicity about the implementation of addiction consultants in general practice” (communication/marketing), “sharing of positive experiences” (modeling), “enhancement of knowledge about the referral options” (education), “actively creating and strengthening connections with addiction care centers” (service provision), and “having fixed contact persons” (service provision). Two measures focusing on structural changes achieved consensus around high applicability: “reimbursement of extra time per patient” (reimbursement incentive) and “offering an easily accessible consult where patients can go without appointment for advice and treatment” (service provision). No consensus was reached on the following item: “having more financial aid for low-threshold referral options” (incentivization).
- *Q.8. What is needed to improve collaboration with addiction treatment centers*? Consensus was achieved on the applicability of eight items to improve collaboration with addiction care centers: “shortening of waiting lists in addiction care centers” (service provision), “faster communication and accessibility to addiction care settings” (service provision), “telephone and online consultations with addiction care settings” (service provision), “financial reimbursements from health insurance companies for better cooperation with addiction care centers” (incentivization), “trainings organized by addiction care centers to improve informal contacts” (service provision), “faster feedback from addiction care centers about patient information” (service provision), and “composing a cooperation protocol with task descriptions” (guideline). No consensus was reached on the following item: “deploying an addiction prevention expert in general practice” (service provision).
- *Q.9. What is needed to make the subject* “*alcohol use*” *easier to discuss in general practice*? “More awareness about attitudes regarding discussing alcohol use with patients” (education), “increasing knowledge and skills” (education), “displaying posters and information in the waiting room about responsible alcohol use” (communication/marketing), “exchanging positive experiences with colleagues about discussing alcohol use with patients” (education/modeling), and “discussing alcohol on the basis of various physical, social, or psychological signs of risky drinking” (regulation) were listed as ways to make the subject easier to discuss. “Supportive materials such as practical tools (e.g., screening instruments or protocols)” (enablement) and “the use of online screening tools” (enablement) were consensually endorsed as well. All items were rated high on applicability, and consensus was reached on all except “asking every patient about alcohol use” (regulation) and “awareness of own alcohol use and not letting this be a reason to avoid discussing alcohol use with patients” (education).
- *Q.10. How applicable do you find the following methods of screening*? Two out of seven items showed consensus and a high degree of applicability: (1) one item endorsed a screening method where patients are asked about alcohol use when they present specific symptoms, such as high blood pressure or gastrointestinal symptoms which might be related to problem drinking; (2) another item endorsed a screening method in which patient groups such as diabetics or obstructive pulmonary disease patients are all screened during periodic checkups. A low degree of applicability and no consensus was reached on any other screening method such as universal screening, self-screening at home, or screening in waiting rooms of general practices.

### Differences Between Groups

Overall, the group disagreed (IQR ≥ 1) on the applicability of 22 items. The Kruskal–Wallis test was performed to identify whether the three main groups: GPs, practice nurses (PNs), and addiction prevention workers (APWs) significantly differed in opinion on these items. We formed three comparison groups (GP-PN, GP-APW, PN-APW) on which a Bonferroni adjustment for multiple comparisons was applied. The three groups differed significantly on ten items (Table 3). Differences were primarily found between GPs and PNs compared to APWs (Table 4) on items reflecting screening methods (screening of all patients, screening of risk groups), service provision strategies (deploying an addiction consultant to improve knowledge and collaboration; an alcohol consultation to have more time to discuss alcohol use), financial incentive strategies (financial aid for low-threshold referral options), enablement strategies (an app with information about ASBI to increase knowledge; distribution of self-report questionnaires by receptionists), and training strategies (training focusing on discussing alcohol use of GPs and PNs to eliminate personal reference frames). On all differing items, GPs and PNs had a significantly lower mean rank than APWs. Lastly, comparing GPs and PNs, PNs had a significantly higher mean rank than GPs on items related to universal screening.

**Table 3:** Kruskal–Wallis test on nonconsented items (*n* = 144)

	Kruskal–Wallis test			
	Mdn	IQR	*H*	*P* value
*Q.1. What is needed to increase knowledge*?				
An app with information about ASBI	5	2	21.07	0.002*
Involving an addiction consultant in general practice	6	2	21.07	0.000*
*Q.2. What is needed to discuss alcohol use independent from personal reference frames*?				
Discussing alcohol use of GPs and PNs in training	5	2	9.69	0.008*
Information about alcohol use in own profession	5	2	3.26	0.196
*Q.3. What is needed to increase motivation to work with problematic alcohol users*?				
Financial incentives for ASBI	5	2	2.83	0.242
Trust between health professional and patient	5	2	5.18	0.075
*Q.4. Which incentives are needed*?				
Insight into financial profits of ASBI	5	1.75	4.30	0.117
Faster referral and treatment—primary/secondary care	6	2	3.56	0.168
A fee of a few euros per patient screened	5	2	.16	0.922
*Q.5. What is the role of the GP*/*PN*?				
The practice nurse specialized in somatic care has an important role in brief treatment of problematic alcohol use	5	3	1.37	0.505
*Q.6. What is needed to implement ASBI despite a lack of time*?				
Distribution of self-report questionnaires by receptionists	4	2	14.92	0.001*
An online program for diagnosing, monitoring, care indication and treatment plans	5	2	2.49	0.228
An alcohol-consultation with more time to discuss alcohol use with patients	5	2	20.94	0.000*
Financial aid for conducting ASBI	5	2	2.93	0.231
*Q.7. What is needed to utilize low*-*threshold referral options*?				
Financial aid for low-threshold referral possibilities	5.5	2	7.10	0.029*
*Q.8. What is needed to improve collaboration*?				
Deploying an addiction prevention expert	6	2	20.09	0.000*
*Q.9. What is needed to make* “*alcohol use*” *easier to discuss*?				
Asking every patient about alcohol use, routinely	6	2	25.60	0.000*
*Q.10. How applicable do you find the following methods of screening*?				
Screening of all patients	4	3	20.56	0.000*
Screening of newly registered patients	6	2	3.72	0.156
Screening of patient risk-groups (e.g., patients above 50 years of age)	6	2	8.57	0.014*
Self-screening by patients in waiting room	4	2	1.98	0.372
Self-screening by patients by means of an online program	5	2	.56	0.756

*P < .05

**Table 4:** Post hoc Mann–Whitney *U* test comparing groups

	GP		PN		APW		GP-PN		GP-APW		PN-APW	
	Mdn	IQR	Mdn	IQR	Mdn	IQR	*Z*	*P*	*Z*	*P*	*Z*	*P*
*Q.1. What is needed to increase knowledge*?												
An app with information about ASBI	5	1	6	1	6	1.25	−3.26	0.001*	−2.79	0.005*	−0.23	0.821
Involving an addiction consultant in general practice	5	2	6	1	7	1	−0.76	0.449	−3.93	0.000*	−4.32	0.000*
*Q.2. What is needed to discuss alcohol use independent from personal reference frames*?												
Discussing alcohol use of GPs and PNs in training	4	2	5	2	6	1	−0.56	0.577	−2.77	0.006*	−2.80	0.005*
*Q.6. What is needed to implement ASBI despite a lack of time*?												
Distribution of self-report questionnaires by receptionists	3	2.5	4	2	5	2	−1.59	0.111	−3.67	0.000*	−2.93	0.003*
An alcohol-consultation with more time to discuss alcohol use with patients	5	3	5	2.5	6	2	−0.86	0.391	−4.23	0.000*	−3.99	0.000*
*Q.7. What is needed to utilize low*-*threshold referral options*?												
Financial aid for low-threshold referral options	5	2	5	2	6	1	−0.35	0.725	−1.99	0.046	−2.64	0.008*
*Q.8. What is needed to improve collaboration*?												
Deploying an addiction prevention expert	6	2	5	2	6.5	1	−1.57	0.117	−2.71	0.007*	−4.48	0.000*
*Q.9. What is needed to make* “*alcohol use*” *easier to discuss*?												
Asking every patient about alcohol use, routinely	4	4	6	2	6	2	−4.76	0.000*	−3.91	0.000*	−0.10	0.920
*Q.10. How applicable do you find the following methods of screening*?												
Screening of all patients	2	2	4	3	5	2	−2.99	0.000*	−4.13	0.000*	−2.70	0.007*
Screening of patient risk groups (e.g., patients above 50 years of age)	6	1	6	1	6.5	1.25	−0.64	0.522	−2.15	0.032	−2.85	0.004*

*P < .016 (adjusted for multiple comparisons)

## Discussion

The current study aimed to identify applicable strategies to overcome barriers and facilitate implementation of ASBI in general practices in the Netherlands. A broad range of strategies was identified that represent views from healthcare professionals and addiction prevention experts and, as such, fit with their beliefs about what is required to implement ASBI in primary healthcare in the Netherlands. On the whole, there was a high level of consensus within and between expert groups about the applicability of strategies to implement ASBI. Nevertheless, there were items which had relatively low median scores on applicability and which did not reach consensus. Therefore, this study managed to show discrimination between items which is in line with the goal of the Delphi method.

Even though there is more acknowledgement for fitting ASBI to the local context (McCormick et al. 2010), the extent to which ASBI may be implemented to fit into clinical practice in a way that can retain its efficacy is currently unclear and highlights the problem of translating ideal-world efficacy trial results into clinically relevant implementation-effectiveness results (Saitz 2014). It is important to ensure that in translating effective interventions to fit into clinical practice, the core effective ingredients of ASBI are retained and not adapted to such an extent that they lose effectiveness (Michie et al. 2012). Additionally, it is equally important to translate the effective ingredients of the implementation process, as seen in efficacy trials, to fit clinical practice as well. For instance, is extensive face-to-face training, as seen in efficacy trials, needed to elicit behavior change in GPs, or is an E-learning program sufficient? The same holds for the method of screening. Is universal screening a core effective ingredient of ASBI? Universal screening has been shown to lead to a higher amount of detection, but is generally considered to be more effortful and time consuming (Beich et al. 2003). GPs seem to be reluctant to discuss drinking unless signs of risk are apparent, which corresponds to a targeted or symptom-specific approach (Reinholdz et al. 2011). This is in line with findings in the current study. The lack of consensus on items related to universal screening reflects the resistance towards these methods, while consensus on the item “bringing up the question of alcohol on the basis of physical, social, or psychological signs” endorsed the value of symptom-specific screening. Additionally, our study also showed that in contrast to GPs, practice nurses are in fact more likely to be willing to screen universally.

A previous Delphi study by Heather et al. (2004), which investigated how ASBI can be implemented in general practices in the UK stressed the need for increased and improved training and education of health professionals regarding recognition of risk factors and brief intervention skills. While there was no consensus in their survey as to the best methods for effective training and education, our study identified applicable educational methods to increase knowledge and awareness such as E-learning, face-to-face training, peer-to-peer coaching, and the use of supportive materials. Some identified educational strategies in our study have not been investigated before and should be taken into account in future ASBI implementation programs. For instance, E-learning technologies in general practices might offer health professionals individualized content tailored to their needs and enhance health professionals’ interactions with others. Internet-based learning has been proven to be at least as effective as traditional learning methods (e.g., lectures) (Ruiz et al. 2006). It enables health professionals to participate at a time and place convenient to them and may therefore provide a more fitting alternative to extensive training programs which might require too much time and effort (Cook et al. 2008; Kulier et al. 2009). However, most implementation trials so far have relied on traditional learning methods.

The views of our study participants showed broad agreement on the applicability of strategies targeting health professionals’ perceptions of ASBI evidence strength. This is in line with findings of Wilson et al. (2011) which show that understanding the evidence-base supporting ASBI could facilitate the implementation of ASBI in everyday primary care practice. The endorsement of these items also reflects the underlying issue of problematic alcohol use having a negative connotation and being associated with universal stigma and marginalization (Room 2005). Experts in our study consensually agreed about the importance of destigmatization and the role of the media to create awareness about the importance of addressing problematic alcohol use.

Although financial incentives for ASBI implementation have been suggested to be potentially effective (Keurhorst et al. 2013), fee-for-service incentives (e.g., monetary fees per screened patient) received little consensual support in our study. However, there was a preference for reimbursement incentives such as reimbursement of extra time per patient and reimbursements from health insurance companies for cooperation with addiction care centers.

GPs and practice nurses agreed on their important roles in the delivery of screening and various forms of brief interventions. Although screening can be done by both nurses and GPs, treatment is generally seen to be part of GPs’ and nurses’ roles. Interestingly, there was no consensus about involving an addiction consultant in the GP setting. In contrast to our findings, the previous Delphi study conducted by Heather et al. (2004) has found strong support for the employment of a specialist alcohol worker to assist in the delivery of ASBI. This might have to do with the fact that, in the Netherlands, the nationwide implementation of the mental health practice nurse in recent years might already have provided enough opportunity for consultation and delivery of more complex alcohol counseling (Van Orden et al. 2009).

A wide variety of strategies have been identified in this study and these can be categorized in one of the intervention function or policy measures as indicated in the BCW. Most strategies identified in this study can be classified in the following intervention functions: (1) education, (2) training, (3) environmental restructuring, (4) enablement, (5) specific types of incentivization, (6) modeling, (7) persuasion, and in the following policy measures: (8) guidelines, (9) regulation, (10) service provision, and (11) communication/marketing. When developing an implementation intervention, suitable strategies from these categories can be selected and brought together as a comprehensive implementation program. This is the first study to provide a systematic and comprehensive overview of applicable strategies for ASBI implementation in the Netherlands. Following the BCW approach, the current study clearly points to the relevance of applying a broad, multifaceted approach to ASBI implementation in general practices. The challenge of developing a multifaceted implementation program that targets the most important barriers and uses potentially effective and applicable strategies underscores the need to use theories as a basis for any future implementation program to make the underlying mechanisms which contribute to the implementation of ASBI explicit and to identify “core components” of the strategies (Grol et al. 2013; Nilsen 2015).

Some limitations of this study should be noted. First, this study did not include policymakers among the experts, which could have provided more insight into other policy measures as potential strategies. Nevertheless, a broad range of topics including policy strategies were covered, which was in line with the aim of this study. Second, this research was based in the clinical context of the Netherlands and may not generalize to other countries. For instance, the lack of consensus about involving an addiction consultant in the GP setting might be due to the nationwide implementation of the mental health practice nurse in the Netherlands, which already provides more opportunity for consultation and ASBI delivery. Yet, this strategy might be beneficial in other countries’ clinical context. Other strategies, such as the use of E-learning technology, incentivization, supportive materials, and more publicity might be considered country-wide strategies. However, this body of research stresses the importance of adapting strategies to country-specific and local needs of primary healthcare professionals. Third, response rates were suboptimal, although they are comparable to response rates in other Delphi studies (Elfeddali et al. 2010; Maijala et al. 2016).

Nevertheless, this exploratory study identified a broad set of intervention and policy strategies aimed at overcoming barriers to ASBI implementation in general practice and paves the way for future research to experimentally test the identified implementation and policy strategies using multifaceted approaches. Based on the findings, the main recommendations for prevention scientists working to expand the use of ASBI programs to support implementation, aligned with the views of the Dutch professionals, are as follows: (1) use of E-learning technology, (2) perform symptom-specific screening by GPs and/or perform universal screening by practice nurses, (3) use of reimbursement incentives, (4) use of supportive materials, (5) clear guidelines, (6) service provision of addiction care centers by offering telephone/online consultations, improving communication with general practices and shortening of waiting lists, and (7) more publicity in the media to create awareness about problematic alcohol use, within and outside the GP setting.

## Electronic supplementary material

Below is the link to the electronic supplementary material.

ESM 1 (DOCX 51 kb)

## References

[CR1] Anderson P, Laurant M, Kaner E, Wensing M, Grol R (2004). Engaging general practitioners in the management of hazardous and harmful alcohol consumption: Results of a meta-analysis. Journal of Studies on Alcohol.

[CR2] Angus, C., Scafato, E., Ghirini, S., Torbica, A., Ferre, F., Struzzo, P., . . . Brennan, A. (2014). Cost-effectiveness of a programme of screening and brief interventions for alcohol in primary care in Italy. *BMC Family Practice, 15*, 26. doi: 10.1186/1471-2296-15-26. 10.1186/1471-2296-15-26 PMC3936801 24502342

[CR3] Beich A, Thorsen T, Rollnick S (2003). Screening in brief intervention trials targeting excessive drinkers in general practice: Systematic review and meta-analysis. British Medical Journal.

[CR4] Bertholet N, Daeppen JB, Wietlisbach V, Fleming M, Burnand B (2005). Reduction of alcohol consumption by brief alcohol intervention in primary care: Systematic review and meta-analysis. Archives of Internal Medicine.

[CR5] Boomsma, L. J., Drost, I. M., Larsen, I. M., Luijkx, J. J. H. M., Meerkerk, G. J., Valken, N., . . . Sijbom, M. (2014). General practice guideline for problematic alcohol use [in Dutch: NHG-Standaard Problematisch alcoholgebruik (Derde herziening)]. *Huisarts en Wetenschap, 57*, 638–646.

[CR6] Burgering E, Willems H (2013). Doctor doesn’t ask enough about alcohol use [in Dutch: Arts vraagt te weinig naar drankgebruik]. Dokter Patient.

[CR7] Cook DA, Levinson AJ, Garside S, Dupras DM, Erwin PJ, Montori VM (2008). Internet-based learning in the health professions: A meta-analysis. The Journal of the American Medical Association.

[CR8] Damschroder LJ, Hagedorn HJ (2011). A guiding framework and approach for implementation research in substance use disorders treatment. Psychology of Addictive Behaviors.

[CR9] Elfeddali I, Bolman C, Mesters I, Wiers RW, de Vries H (2010). Factors underlying smoking relapse prevention: Results of an international Delphi study. Health Education Research.

[CR10] Funk, M., Wutzke, S., Kaner, E., Anderson, P., Pas, L., McCormick, R., . . . World Health Organization Brief Intervention Study Group. (2005). A multicountry controlled trial of strategies to promote dissemination and implementation of brief alcohol intervention in primary health care: Findings of a World Health Organization collaborative study. *Journal of Studies on Alcohol, 66*(3), 379–388. 10.15288/jsa.2005.66.379 16047527

[CR11] Grol R, Wensing M, Eccles M, Davis D (2013). Improving patient care: The implementation of change in health care.

[CR12] Heather N, Dallolio E, Hutchings D, Kaner E, White M (2004). Implementing routine screening and brief alcohol intervention in primary health care: A Delphi survey of expert opinion. Journal of Substance Use.

[CR13] Huijg JM, Crone MR, Verheijden MW, van der Zouwe N, Middelkoop BJ, Gebhardt WA (2013). Factors influencing the adoption, implementation, and continuation of physical activity interventions in primary health care: A Delphi study. BMC Family Practice.

[CR14] Johnson M, Jackson R, Guillaume L, Meier P, Goyder E (2011). Barriers and facilitators to implementing screening and brief intervention for alcohol misuse: A systematic review of qualitative evidence. Journal of Public Health (Oxford).

[CR15] Kaner EF (2010). NICE work if you can get it: Development of national guidance incorporating screening and brief intervention to prevent hazardous and harmful drinking in England. Drug and Alcohol Review.

[CR16] Kaner, E. F., Dickinson, H. O., Beyer, F., Pienaar, E., Schlesinger, C., Campbell, F., . . . Heather, N. (2009). The effectiveness of brief alcohol interventions in primary care settings: A systematic review. *Drug and Alcohol Review, 28*, 301–323. doi: 10.1111/j.1465-3362.2009.00071.x 10.1111/j.1465-3362.2009.00071.x 19489992

[CR17] Keurhorst, M. N., Anderson, P., Spak, F., Bendtsen, P., Segura, L., Colom, J., . . . Laurant, M. G. H. (2013). Implementing training and support, financial reimbursement, and referral to an internet-based brief advice program to improve the early identification of hazardous and harmful alcohol consumption in primary care (ODHIN): Study protocol for a cluster randomized factorial trial. *Implementation Science, 8,* 11. 10.1186/1748-5908-8-11 PMC3564747 23347874

[CR18] Kulier, R., Coppus, S. F., Zamora, J., Hadley, J., Malick, S., Das, K., . . . Khan, K. S. (2009). The effectiveness of a clinically integrated e-learning course in evidence-based medicine: A cluster randomised controlled trial. *BMC Medical Education, 9*, 21. doi: 10.1186/1472-6920-9-21 10.1186/1472-6920-9-21 PMC2688004 19435520

[CR19] Lean ME, Mann JI, Hoek JA, Elliot RM, Schofield G (2008). Translational research. BMJ.

[CR20] Lim, S. S., Vos, T., Flaxman, A. D., Danaei, G., Shibuya, K., Adair-Rohani, H., . . . Memish, Z. A. (2012). A comparative risk assessment of burden of disease and injury attributable to 67 risk factors and risk factor clusters in 21 regions, 1990–2010: A systematic analysis for the Global Burden of Disease Study 2010. *Lancet, 380*, 2224–2260. doi: 10.1016/S0140-6736(12)61766-8 10.1016/S0140-6736(12)61766-8 PMC4156511 23245609

[CR21] Linstone HA, Turoff M (1975). The Delphi method: Techniques and applications.

[CR22] Maijala V, Tossavainen K, Turunen H (2016). Health promotion practices delivered by primary health care nurses: Elements for success in Finland. Applied Nursing Research.

[CR23] McCormick, R., Docherty, B., Segura, L., Colom, J., Gual, A., Cassidy, P., . . . Heather, N. (2010). The research translation problem: Alcohol screening and brief intervention in primary care - Real world evidence supports theory. *Drugs-Education Prevention and Policy, 17*, 732–748. doi: 10.3109/09687630903286800

[CR24] Michie S, van Stralen MM, West R (2011). The behaviour change wheel: A new method for characterising and designing behaviour change interventions. Implementation Science.

[CR25] Michie S, Whittington C, Hamoudi Z, Zarnani F, Tober G, West R (2012). Identification of behaviour change techniques to reduce excessive alcohol consumption. Addiction.

[CR26] Nilsen P (2015). Making sense of implementation theories, models and frameworks. Implementation Science.

[CR27] Purshouse, R. C., Brennan, A., Rafia, R., Latimer, N. R., Archer, R. J., Angus, C. R., . . . Meier, P. S. (2013). Modelling the cost-effectiveness of alcohol screening and brief interventions in primary care in England. *Alcohol Alcohol, 48*, 180–188. doi: 10.1093/alcalc/ags103 10.1093/alcalc/ags103 23015608

[CR28] Reinholdz HK, Bendtsen P, Spak F (2011). Different methods of early identification of risky drinking: A review of clinical signs. Alcohol and Alcoholism.

[CR29] Room R (2005). Stigma, social inequality and alcohol and drug use. Drug and Alcohol Review.

[CR30] Ruiz JG, Mintzer MJ, Leipzig RM (2006). The impact of E-learning in medical education. Acadamic Medicine.

[CR31] Saitz R (2014). The best evidence for alcohol screening and brief intervention in primary care supports efficacy, at best, not effectiveness: you say tomato, I say tomato? That’s not all it’s about. Addiction Science and Clinical Practice.

[CR32] Van Beurden I, Anderson P, Akkermans RP, Grol RPTM, Wensing M, Laurant MGH (2012). Involvement of general practitioners in managing alcohol problems: A randomized controlled trial of a tailored improvement programme. Addiction.

[CR33] Van Dijck D, Knibbe RA (2005). The prevalance of problematic alcohol use in the Netherlands [in Dutch: De Prevalentie van Probleemdrinken in Nederland].

[CR34] Van Orden M, Hoffman T, Haffmans J, Spinhoven P, Hoencamp E (2009). Collaborative mental health care versus care as usual in a primary care setting: A randomized controlled trial. Psychiatric Services.

[CR35] Wensing, M., Huntink, E., van Lieshout, J., Godycki-Cwirko, M., Kowalczyk, A., Jager, C., . . . Baker, R. (2014). Tailored implementation of evidence-based practice for patients with chronic diseases. *PLoS One, 9*, e101981. doi: 10.1371/journal.pone.0101981 10.1371/journal.pone.0101981 PMC4087017 25003371

[CR36] Wilson GB, Lock CA, Heather N, Cassidy P, Christie MM, Kaner EF (2011). Intervention against excessive alcohol consumption in primary health care: A survey of GPs’ attitudes and practices in England 10 years on. Alcohol and Alcoholism.

[CR37] World Health Organization. (2014). *Global status report on alcohol and health 2014*. Retrieved from http://apps.who.int/iris/bitstream/10665/112736/1/9789240692763_eng.pdf.

