# Supplementary material for: Strategies to Overcome Barriers to Implementation of Alcohol Screening and Brief Intervention in General Practice: a Delphi Study Among Healthcare Professionals and Addiction Prevention Experts
Source: Prev Sci. 2016 May 11;17:689–99. doi: 10.1007/s11121-016-0653-4 (PMC4938847; doi:10.1007/s11121-016-0653-4)
Supplement: Supplementary file 1 — (DOCX 51 kb) [file 11121_2016_653_MOESM1_ESM.docx]

**Supplementary table**

|  | | **Total sample** | | | **GPs** | | | **PNs** | | | **APWs** | | |
| --- | --- | --- | --- | --- | --- | --- | --- | --- | --- | --- | --- | --- | --- |
| ***Capability – What is needed to increase knowledge about symptoms, risk groups and intervention techniques?*** | | ***N*** | **Mdn** | **IQR** | ***N*** | **Mdn** | **IQR** | ***N*** | **Mdn** | **IQR** | ***N*** | **Mdn** | **IQR** |
| **Intervention functions and policies (BCW)** | **Proposed intervention** |  |  |  |  |  |  |  |  |  |  |  |  |
| Education | E-learning | 183 | 6 | 2 | 52 | 6 | 1 | 82 | 6 | 1 | 43 | 6 | 2 |
| Education | Learning through examples and insight into favorable results of ASBI | 183 | 6 | 1 | 52 | 5 | 1 | 82 | 6 | 2 | 43 | 6 | 1 |
| Enablement | Website with information about how to implement ASBI in general practice | 183 | 6 | 1 | 52 | 5 | 1 | 82 | 6 | 2 | 43 | 6 | 1 |
| Enablement | An app with information about how to conduct ASBI | 182 | 5 | 2 | 51 | 5 | 3 | 82 | 5 | 2 | 43 | 6 | 1 |
| Service provision | Involving an addiction consultant in the general practice | 182 | 6 | 2 | 51 | 5 | 2 | 82 | 5 | 2 | 43 | 7 | 1 |
| Service provision | Involving addiction centers in the organization of information gathering | 182 | 6 | 2 | 51 | 6 | 1 | 82 | 6 | 1.25 | 43 | 7 | 1 |
| Training | Face-to-face expertise-enhancement training | 183 | 6 | 1 | 52 | 6 | 1 | 82 | 6 | 1 | 43 | 7 | 1 |
| Communication/marketing | More attention and publicity about problematic alcohol use in the media and in the general practice setting | 182 | 6 | 1 | 51 | 6 | 1 | 82 | 6 | 1 | 43 | 6 | 1 |
| ***Capability – What is needed to discuss alcohol use with patients independent from reference frames formed by own alcohol use?*** | |  |  |  |  |  |  |  |  |  |  |  |  |
| **Intervention functions and policies (BCW)** | **Proposed intervention** |  |  |  |  |  |  |  |  |  |  |  |  |
| Training | Peer-to-peer coaching about professional attitude to become more aware of own behavior, drinking norms and reference frames | 179 | 6 | 1 | 50 | 5 | 2 | 80 | 5 | 1 | 43 | 6 | 1 |
| Training | Discussing alcohol use of GPs and PNs in training (e.g. by means of a self-test) | 178 | 5 | 2 | 50 | 5 | 1.25 | 79 | 5 | 2 | 43 | 6 | 1 |
| Education | Information about alcohol use of GPs and PNs | 179 | 5 | 2 | 50 | 5 | 2 | 80 | 5 | 2 | 43 | 6 | 2 |
| Guidelines | Clear and standardized guidelines, protocols and norms about ASBI | 179 | 6 | 1 | 50 | 5 | 2 | 80 | 6 | 1 | 43 | 6 | 2 |
| Communication/marketing | Destigmatization of problematic alcohol use | 179 | 6 | 1 | 50 | 5 | 2 | 80 | 6 | 1 | 43 | 6 | 2 |
| ***Motivation – What is needed to increase motivation to work with problematic alcohol users?*** | |  |  |  |  |  |  |  |  |  |  |  |  |
| **Intervention functions and policies (BCW)** | **Proposed intervention** |  |  |  |  |  |  |  |  |  |  |  |  |
| Education | Knowledge about how to work with problematic alcohol users | 179 | 6 | 1 | 50 | 6 | 1 | 80 | 6 | 1 | 43 | 6 | 1 |
| Education | Knowledge about how symptoms are associated with problematic alcohol use | 180 | 6 | 1 | 50 | 6 | 1 | 81 | 6 | 1 | 43 | 7 | 1 |
| Education | Knowledge about the effectiveness of ASBI | 180 | 6 | 1 | 50 | 6 | 1 | 81 | 6 | 1 | 43 | 6 | 2 |
| Education | Clear instructions for treatment | 180 | 6 | 2 | 50 | 6 | 1 | 81 | 6 | 1 | 43 | 6 | 2 |
| Education | Learning to establish trust between health professional and patient | 179 | 5 | 2 | 49 | 4 | 2 | 81 | 5 | 2 | 43 | 5 | 2 |
| Enablement | Practical tools for patients (e.g. alcohol diary or agenda) | 180 | 6 | 1 | 50 | 5 | 1 | 81 | 6 | 0.5 | 43 | 6 | 1 |
| Enablement | Distinguishing problematic alcohol users from dependent drinkers | 180 | 6 | 1.75 | 50 | 6 | 1 | 81 | 6 | 2 | 43 | 6 | 2 |
| Service provision | More accessible referral options and consultations with experts for support and cooperation | 180 | 6 | 1 | 50 | 6 | 1 | 81 | 6 | 2 | 43 | 7 | 1 |
| Incentivization | Financial incentives for ASBI | 180 | 5 | 2 | 50 | 4 | 2.25 | 81 | 5 | 2 | 43 | 5 | 2 |
| Communication/marketing,  Education | More publicity about the possibilities of ASBI by means of E-health | 180 | 6 | 1 | 50 | 6 | 1 | 81 | 6 | 1 | 43 | 6 | 2 |
| ***Motivation – Which incentives are needed to implement ASBI effectively in routine practice?*** | |  |  |  |  |  |  |  |  |  |  |  |  |
| **Intervention functions and policies (BCW)** | **Proposed intervention** |  |  |  |  |  |  |  |  |  |  |  |  |
| Persuasion | Insight into the financial profits of ASBI | 179 | 5 | 2 | 50 | 5 | 2 | 80 | 5 | 2 | 43 | 6 | 2 |
| Persuasion | Insight into the health profits of ASBI | 179 | 6 | 1 | 50 | 6 | 1 | 80 | 6 | 2 | 43 | 7 | 1 |
| Incentivization | Financial contributions to projects in general practice about ASBI | 179 | 6 | 1 | 50 | 6 | 1.25 | 80 | 6 | 1.75 | 43 | 6 | 2 |
| Incentivization | Financial reimbursements from health insurance companies to implement ASBI | 179 | 6 | 1 | 50 | 5 | 2 | 80 | 6 | 1 | 43 | 6 | 2 |
| Incentivization | A monetary fee per screened patient | 179 | 5 | 2 | 50 | 4 | 2 | 80 | 5 | 2 | 43 | 5 | 2 |
| Service provision | Implementing a practice nurse specialized in addiction problems without extra costs | 178 | 6 | 2 | 50 | 6 | 1.25 | 79 | 6 | 2 | 43 | 7 | 1 |
| Service provision | A faster referral and treatment in primary care and secondary care | 179 | 6 | 2 | 50 | 6 | 1.25 | 80 | 6 | 2 | 43 | 7 | 2 |
| ***Motivation – What is the professional role of the GP/practice nurse in ASBI?*** | |  |  |  |  |  |  |  |  |  |  |  |  |
| The GP has an important role in early detection of problematic alcohol use | | 214 | 6 | 1 | 60 | 6 | 1 | 95 | 6 | 1 | 50 | 7 | 1 |
| The GP has an important role in brief treatment of problematic alcohol use by means of brief advice and monitoring | | 213 | 6 | 1 | 60 | 6 | 0 | 95 | 6 | 1 | 49 | 6 | 1 |
| The GP has an important role in brief treatment of problematic alcohol use by means of Motivational Interviewing | | 211 | 6 | 1 | 59 | 6 | 1 | 94 | 5 | 1 | 49 | 5 | 2 |
| The practice nurse mental health has an important role in early detection of problematic alcohol use | | 211 | 6 | 1 | 59 | 6 | 2 | 95 | 6 | 1 | 49 | 7 | 1 |
| The practice nurse mental health has an important role in brief treatment of problematic alcohol use | | 209 | 6 | 1 | 59 | 6 | 1 | 94 | 6 | 1 | 48 | 6.5 | 1 |
| The practice nurse somatic care has an important role in early detection of problematic alcohol use | | 207 | 6 | 1 | 58 | 6 | 1 | 94 | 6 | 1 | 47 | 7 | 1 |
| The practice nurse somatic care has an important role in brief treatment of problematic alcohol use | | 205 | 5 | 2 | 58 | 5 | 3 | 92 | 5 | 2 | 47 | 5 | 2 |
| ***Opportunity – What is needed to implement ASBI in routine care despite lack of time?*** | |  |  |  |  |  |  |  |  |  |  |  |  |
| **Intervention functions and policies (BCW)** | **Proposed intervention** |  |  |  |  |  |  |  |  |  |  |  |  |
| Enablement | Distribution of self-report questionnaires by receptionists in the waiting room before consultation | 179 | 6 | 1 | 50 | 3 | 3 | 80 | 4 | 3 | 43 | 5 | 3 |
| Enablement | A short and simple screening instrument such as the AUDIT-C | 179 | 6 | 2 | 50 | 6 | 1.25 | 80 | 6 | 1.75 | 43 | 7 | 1 |
| Enablement | Adding a question about alcohol to a lifestyle questionnaire that is frequently used (e.g. Four Dimensional Symptom Questionnaire; 4DSQ) | 179 | 6 | 1 | 50 | 6 | 1 | 80 | 6 | 1 | 43 | 7 | 1 |
| Enablement | A self-report questionnaire for patients | 179 | 6 | 1 | 50 | 6 | 1 | 80 | 6 | 1 | 43 | 5 | 1 |
| Regulation | More time per consultation | 179 | 6 | 1 | 50 | 6 | 1 | 80 | 6 | 2 | 43 | 6 | 2 |
| Regulation | Scheduling a second appointment | 179 | 6 | 1 | 50 | 6 | 1 | 80 | 6 | 0.75 | 43 | 6 | 1 |
| Environmental restructuring | An online program for diagnosing, monitoring, care indication and treatment plans | 179 | 5 | 2 | 50 | 5 | 4 | 80 | 5 | 2 | 43 | 5 | 2 |
| Environmental restructuring | A short questionnaire (e.g. 5-shot questionnaire) in the registration system of the general practice | 179 | 6 | 1 | 50 | 6 | 2 | 80 | 6 | 1 | 43 | 6 | 2 |
| Education | Knowledge about the time-efficiency of ASBI | 179 | 6 | 1 | 50 | 6 | 0 | 80 | 6 | 0.75 | 43 | 7 | 1 |
| Service provision | An alcohol consultation with more time to discuss alcohol use with patients | 179 | 5 | 2 | 50 | 5 | 3 | 80 | 5 | 2 | 43 | 6 | 2 |
| Incentivization | Financial aid for conducting ASBI | 179 | 5 | 2 | 50 | 5 | 2 | 80 | 5 | 2 | 43 | 6 | 3 |
| ***Opportunity – What is needed to utilize low-threshold referral options in general practice?*** | |  |  |  |  |  |  |  |  |  |  |  |  |
| **Intervention functions and policies (BCW)** | **Proposed intervention** |  |  |  |  |  |  |  |  |  |  |  |  |
| Service provision | Creating and strengthening connections with addiction care centers | 179 | 6 | 1 | 50 | 6 | 0.25 | 80 | 6 | 1 | 43 | 7 | 0 |
| Service provision | A fixed contact person within addiction care centers | 179 | 6 | 1 | 50 | 6 | 1 | 80 | 6 | 1 | 43 | 7 | 1 |
| Service provision | An easily accessible consultation where patients can go without appointment for advice and treatment | 179 | 6 | 1 | 50 | 6 | 2 | 80 | 6 | 1 | 43 | 6 | 2 |
| Education | Enhancing knowledge about the availability of accessible and low-threshold referral options | 179 | 6 | 1 | 50 | 6 | 1 | 80 | 6 | 1 | 43 | 7 | 1 |
| Education | Sharing positive experiences about low-threshold referral options | 179 | 6 | 1 | 50 | 6 | 1 | 80 | 6 | 1 | 43 | 6 | 1 |
| Incentivization | Financial aid for low-threshold referral options | 179 | 6 | 2 | 50 | 6 | 2 | 80 | 6 | 1.75 | 43 | 6 | 2 |
| Incentivization | Financial reimbursement of extra time per patient | 179 | 6 | 1 | 50 | 6 | 1.25 | 80 | 6 | 1 | 43 | 6 | 2 |
| Communication/marketing | Information and publicity about the implementation of addiction consultants in general practices | 179 | 6 | 1 | 50 | 6 | 0 | 80 | 6 | 1 | 43 | 7 | 1 |
| ***Opportunity – What is needed to improve collaboration with addiction treatment centres?*** | |  |  |  |  |  |  |  |  |  |  |  |  |
| **Intervention functions and policies (BCW)** | **Proposed intervention** |  |  |  |  |  |  |  |  |  |  |  |  |
| Service provision | Telephone consultations with addiction care centers | 179 | 6 | 1 | 50 | 6 | 2 | 80 | 6 | 1 | 43 | 7 | 1 |
| Service provision | Online consultations with addiction care centers | 179 | 6 | 2 | 50 | 6 | 1 | 80 | 6 | 1.75 | 43 | 6 | 1 |
| Service provision | Faster feedback from addiction care centers about patient information | 178 | 6 | 1 | 50 | 6 | 1.25 | 79 | 6 | 1 | 43 | 7 | 1 |
| Service provision | Deploying an addiction prevention expert in general practice and creating better connections | 178 | 6 | 2 | 50 | 6 | 2 | 79 | 6 | 2 | 43 | 7 | 1 |
| Service provision | Shortening of waiting lists in addiction care centers | 179 | 6 | 1 | 50 | 6 | 1 | 80 | 6 | 1 | 43 | 6 | 2 |
| Enablement | Using an easy registration telephone number for faster communication and accessibility to addiction care centers | 179 | 6 | 1 | 50 | 6 | 2 | 80 | 6 | 1.75 | 43 | 7 | 1 |
| Training | Training organized by addiction care centers to improve informal contacts | 178 | 6 | 1 | 50 | 6 | 1 | 79 | 6 | 1 | 43 | 6 | 1 |
| Incentivization | Financial reimbursements from health insurance companies for better cooperation with addiction care centers | 179 | 6 | 1 | 50 | 6 | 2 | 80 | 6 | 1 | 43 | 6 | 2 |
| Guideline | Composing a cooperation protocol with descriptions of who does what work | 179 | 6 | 1 | 50 | 6 | 1 | 80 | 6 | 1 | 43 | 6 | 1 |
| ***Opportunity – What is needed to make the subject ‘alcohol use’ easier to discuss for health professionals in general practice?*** | |  |  |  |  |  |  |  |  |  |  |  |  |
| **Intervention functions and policies (BCW)** | **Proposed intervention** |  |  |  |  |  |  |  |  |  |  |  |  |
| Education | Exchanging positive experiences with colleagues about discussing alcohol use with patients | 186 | 6 | 2 | 54 | 5 | 2 | 83 | 6 | 1 | 43 | 7 | 1 |
| Education | Increasing knowledge and skills of GPs and practice nurses | 188 | 6 | 1 | 55 | 6 | 0 | 82 | 6 | 1 | 44 | 7 | 1 |
| Education | Increasing GPs’ and Practice nurses’ awareness of own attitude | 186 | 6 | 1 | 54 | 6 | 1 | 83 | 6 | 1 | 43 | 7 | 1 |
| Education | Increasing GPs’ and Practice nurses’ awareness of own alcohol use | 186 | 6 | 2 | 54 | 6 | 2 | 83 | 6 | 1 | 43 | 7 | 1 |
| Enablement | Practical/accessible tools (e.g. screening instruments or protocols) | 187 | 6 | 1 | 55 | 6 | 1 | 81 | 6 | 1 | 44 | 6 | 1 |
| Enablement | Online screening questionnaires | 188 | 6 | 1 | 54 | 5 | 2 | 83 | 6 | 1 | 44 | 6 | 2 |
| Regulation | Discussing alcohol use with every patient and mentioning that questions about alcohol use are part of routine care | 187 | 6 | 2 | 54 | 4 | 2.25 | 83 | 6 | 2 | 44 | 6 | 2 |
| Regulation | Discussing alcohol on the basis of various physical, social or psychological signs of risky drinking | 186 | 6 | 1 | 53 | 6 | 1 | 83 | 6 | 1 | 44 | 7 | 1 |
| Communication/marketing | Displaying posters and information in the waiting room about responsible alcohol use | 187 | 6 | 1 | 54 | 5 | 1.25 | 83 | 6 | 1 | 44 | 6 | 2 |
| ***Screening Method – How applicable do you find the following methods of screening?*** | |  |  |  |  |  |  |  |  |  |  |  |  |
| Screening of all patients in general practice | | 200 | 4 | 4 | 57 | 2 | 3.5 | 91 | 4 | 3 | 45 | 5 | 2 |
| Screening of all newly registered patients | | 200 | 5 | 2 | 57 | 4 | 3 | 91 | 5 | 2 | 45 | 6 | 2 |
| Screening of patient groups during periodic check-ups (e.g. diabetics, chronic obstructive pulmonary disease patients) | | 198 | 6 | 1 | 57 | 6 | 0 | 90 | 6 | 2 | 44 | 7 | 1 |
| Screening of patient risk-groups (e.g. patients above 50 years of age, men with psycho-social problems and/or major life events) | | 199 | 6 | 2 | 57 | 5 | 3 | 90 | 6 | 2 | 45 | 6 | 1.5 |
| Symptom-specific screening: inquiring about alcohol when patient present symptoms such as high blood pressure, gastrointestinal symptoms, depression or sleeping problems | | 200 | 6 | 1 | 57 | 6 | 1 | 91 | 6 | 1 | 45 | 7 | 1 |
| Self-screening of patients in waiting rooms using a self-assessment tool | | 198 | 4 | 2 | 57 | 4 | 3 | 89 | 4 | 2.5 | 45 | 5 | 2 |
| Self-screening of patients at home using an online screening program | | 197 | 5 | 2 | 57 | 5 | 3 | 88 | 5 | 2 | 45 | 5 | 2 |
